# Comparative efficacy between real-world and randomized studies of palbociclib+endocrine therapy in HR-positive/HER2-negative metastatic breast cancer: systematic review and meta-analysis

**DOI:** 10.1093/jncics/pkaf083

**Published:** 2025-08-12

**Authors:** Francesco Schettini, Sabrina Nucera, Giuseppe Di Grazia, Fabiola Giudici, Carla Strina, Manuela Milani, Richard Tancredi, Benedetta Conte, Carmen Criscitiello, Mario Giuliano, Matteo Lambertini, Rodrigo Sánchez-Bayona, Tomás Pascual, Grazia Arpino, Lucia Del Mastro, Paolo Vigneri, Massimo Cristofanilli, Hope S Rugo, Alessandra Gennari, Giuseppe Curigliano, Daniele Generali

**Affiliations:** Translational Genomics and Targeted Therapies in Solid Tumors Group, August Pi i Sunyer Biomedical Research Institute (IDIBAPS), Barcelona, Spain; Department of Medical Oncology, Clinic Barcelona Comprehensive Cancer Center, Barcelona, Spain; Faculty of Medicine and Health Sciences, University of Barcelona, Barcelona, Spain; Translational Genomics and Targeted Therapies in Solid Tumors Group, August Pi i Sunyer Biomedical Research Institute (IDIBAPS), Barcelona, Spain; Department of Clinical and Experimental Medicine, University of Catania, Catania, Italy; Translational Genomics and Targeted Therapies in Solid Tumors Group, August Pi i Sunyer Biomedical Research Institute (IDIBAPS), Barcelona, Spain; Department of Human Pathology “G. Barresi”, University of Messina, Messina, Italy; Cancer Epidemiology Unit, Centro di Riferimento Oncologico di Aviano (CRO) IRCCS, Aviano, Italy; Multidisciplinary Unit of Breast Pathology and Translational Research, Cremona Hospital, Cremona, Italy; Multidisciplinary Unit of Breast Pathology and Translational Research, Cremona Hospital, Cremona, Italy; Multidisciplinary Unit of Breast Pathology and Translational Research, Cremona Hospital, Cremona, Italy; Translational Genomics and Targeted Therapies in Solid Tumors Group, August Pi i Sunyer Biomedical Research Institute (IDIBAPS), Barcelona, Spain; Department of Translational Medicine, University of Piemonte Orientale, Novara, Italy; Department of Oncology and Hemato-Oncology, University of Milano, Milan, Italy; Division of Experimental Therapeutics, European Institute of Oncology, IRCCS, Milan, Italy; Department of Clinical Medicine and Surgery, University Federico II, Naples, Italy; Department of Internal Medicine and Medical Specialties (DiMI), School of Medicine, University of Genova, Genova, Italy; Department of Medical Oncology, UOC Clinica di Oncologia Medica, IRCCS Ospedale Policlinico San Martino, Genova, Italy; Department of Medical Oncology, Hospital Universitario, Madrid, Spain; Translational Genomics and Targeted Therapies in Solid Tumors Group, August Pi i Sunyer Biomedical Research Institute (IDIBAPS), Barcelona, Spain; Department of Medical Oncology, Clinic Barcelona Comprehensive Cancer Center, Barcelona, Spain; Faculty of Medicine and Health Sciences, University of Barcelona, Barcelona, Spain; Department of Clinical Medicine and Surgery, University Federico II, Naples, Italy; Department of Internal Medicine and Medical Specialties (DiMI), School of Medicine, University of Genova, Genova, Italy; Department of Medical Oncology, UOC Clinica di Oncologia Medica, IRCCS Ospedale Policlinico San Martino, Genova, Italy; Department of Clinical and Experimental Medicine, University of Catania, Catania, Italy; Medical Oncology Unit, Istituto Clinico Humanitas, Misterbianco, Catania, Italy; Department of Medicine, Division of Hematology-Oncology, Weill Cornell Medicine, New York, NY, United States; Department of Medicine (Hematology/Oncology), University of California San Francisco Helen Diller Family Comprehensive Cancer Center, San Francisco, CA, United States; Department of Translational Medicine, University of Piemonte Orientale, Novara, Italy; Division of Medical Oncology, Maggiore University Hospital, Novara, Italy; Department of Oncology and Hemato-Oncology, University of Milano, Milan, Italy; Division of Experimental Therapeutics, European Institute of Oncology, IRCCS, Milan, Italy; Multidisciplinary Unit of Breast Pathology and Translational Research, Cremona Hospital, Cremona, Italy; Department of Medical, Surgical and Health Sciences, University of Trieste, Trieste, Italy

## Abstract

**Background:**

Cyclin-dependent kinase 4/6 inhibitors (CDK4/6i) combined with endocrine therapy are the standard-of-care for hormone receptor-positive (HR+)/HER2-negative (HER2−) metastatic breast cancer (MBC). Palbociclib, the first approved CDK4/6i, significantly improved progression-free survival (PFS) in randomized controlled trials (RCTs). However, real-world (RW) outcomes may differ due to broader patient populations. This meta-analysis evaluates the applicability of pivotal RCT findings to RW settings.

**Methods:**

We conducted a systematic review and meta-analysis of RW studies on HR+/HER2− MBC treated with palbociclib+aromatase inhibitors (AI) or fulvestrant, reporting median PFS (mPFS) and/or overall survival (mOS). Pooled mPFS/OS was estimated using the median of medians (MM) and weighted MM (WM). RW estimates were deemed comparable to RCTs if MMPFS/OS or WMPFS/OS fell within RCTs’ 95% confidence intervals (CIs). Similar criteria applied to pooled hazard ratios (HRs) of PFS/OS for palbociclib+AI vs AI in visceral/nonvisceral subgroups.

**Results:**

Twelve RW studies were analyzed. First-line palbociclib+AI MMPFS (22.5 months, 95% CI = 19.5 to 31.8) aligned with PALOMA-1/2 pooled mPFS (23.9, 95% CI = 20.2 to 27.6). First-line palbociclib+fulvestrant MMPFS (13.5, 95% CI = 11.6 to 28.5) exceeded PALOMA-3 (11.2, 95% CI = 9.5 to 12.9). Second-line palbociclib+fulvestrant MMPFS (11.5 months, 95% CI = 6.3 to 15.3) was consistent with PALOMA-3. RW first-line mOS (51.2 months, 95% CI = 49.1 to 53.3) surpassed PALOMA-1/2 pooled mOS (45.7, 95% CI = 37.5 to 53.8). WMOS (49.1 months, 95% CI = 49.1 to 53.3) was slightly lower than RCTs (53.7, 95% CI = 37.5 to 53.8). Palbociclib+AI outperformed AI in RW visceral disease, aligning with RCTs, and showed heterogeneous but favorable benefit in nonvisceral disease.

**Conclusions:**

RW data confirm palbociclib+endocrine therapy effectiveness, reinforcing its applicability to broader patient populations.

## Introduction

Breast cancer is the most commonly diagnosed cancer in women globally, and its incidence has been slowly increasing over the past decade.^1^ Across the spectrum of breast cancer subtypes hormone receptor-positive (HR+) and human epidermal growth factor receptor 2-negative (HER2−) represents the most prevalent, accounting for approximately 70% of all cases.^2^ Endocrine therapy (ET) usually represents the upfront treatment for HR+/HER2− metastatic breast cancer (MBC) but its efficacy is limited by the development of various mechanisms of resistance.^3^ Cyclin-dependent kinase 4/6-inhibitors (CDK4/6i), namely, palbociclib, ribociclib, and abemaciclib, combined to ET were able to double median progression-free survival (mPFS) of single-agent ET either in the first-line or second-/further-line setting in all their pivotal phase 3 trials, as well as improving median overall survival (mOS) in some cases.^4^^,^^5^ Consequently, they are now considered the standard of care for de novo or recurrent MBC, either in cases of primary or secondary endocrine resistance.^6-9^ Palbociclib was approved for the first-line treatment of HR+/HER2− MBC in combination with an aromatase inhibitor (AI) for endocrine sensitive MBC or with fulvestrant for endocrine resistant cases. The combination with fulvestrant was also approved for second-/further-lines in patients who have been previously treated with ET in the metastatic setting. These approvals were based on efficacy and tolerability results from the phase 3 PALOMA-2 and PALOMA-3 randomized controlled trials (RCTs), respectively.^10^^,^^11^ Although RCTs are considered the gold standard in clinical research, their results are not always easily generalizable beyond the study population due to usually strict inclusion criteria or highly regulated protocols. Therefore, real-world data provide valuable insights into the efficacy and safety of treatments in routine clinical settings, in a more diverse patient population than RCTs, including individuals with comorbidities or other clinical features that may be underrepresented in clinical trials.^12^ Importantly, real-world data can provide long-term follow-up data, offering insights into the duration of treatment effects and potential late-emerging adverse events that cannot be detected in RCTs, especially if post-progression follow-up is not contemplated in the study protocol. Nonetheless, real-world studies (RWSs) are often subject to biases, such as selection errors and confounding variables, and the quality and completeness of the data can vary between different sources, which can influence the interpretation of the results.^13^^,^^14^

Palbociclib was the first orally bioavailable CDK4/6i to be approved for the clinical practice, and thus is the one for which most real-world evidence has been published so far. The purpose of this meta-analysis was to evaluate the efficacy results of palbociclib in combination with ET in the treatment of HR+/HER2− MBC as observed in RWSs and compare them with the results of RCTs to ensure generalizability to the broader real-life population.

## Methods

### Search strategy, selection criteria, and data extraction

A systematic review of the literature was performed on PubMed to select all RWSs published between 2018 and 2024 including HR+/HER2− pre-/postmenopausal patients with MBC treated with palbociclib+ET (either AI or fulvestrant), in first- and second-/further-lines. The studies could be retrospective or prospective, with 1 or multiple cohorts, comparative or not, but had to report at least median PFS (mPFS) and/or OS (mOS) with their 95% confidence intervals (CIs) for each cohort. The search query is reported in the Supplementary Methods. We also consulted the European Society for Medical Oncology (ESMO), American Society of Clinical Oncology meetings (ASCO), and San Antonio Breast Cancer Symposium (SABCS) annual meetings online databases. The search and data extraction were conducted by 2 investigators (F.G. and F.S.) and a third was consulted in case of controversy (D.G.).

Details about the study design, patient characteristics, interventions, and previous treatments mPFS and/or mOS with 95% confidence interval were extracted from each article, as well as hazard ratios (HRs) for PFS and OS and associated 95% confidence interval, when available.

### Study objectives

The primary objective of our study was to investigate the comparability of the pooled mPFS of RWSs with that of RCTs involving patients with HR+/HER2− MBC treated with palbociclib+AI in the first-line and palbociclib+fulvestrant in the first-line. Secondary objectives included demonstrating that mOS in the same settings were comparable between RWSs and RCTs and that the impact on PFS and OS of palbociclib+AI vs AI in visceral, nonvisceral, and bone-only disease was comparable between RWSs and RCTs. The RCTs considered as reference were the PALOMA-1 and PALOMA-2 for first-line palbociclib+AI and the PALOMA-3 for first-line and second-/further-line palbociclib+fulvestrant.

### Data analysis

Individual patient-level data were not available from any study. The characteristics of the included studies and populations were analyzed in a descriptive manner. The mPFS and mOS extracted from each study cohort were pooled through the method proposed by McGrath et al.,^15^ namely, using the median of medians (MM), and weighted median of medians (WM), with weights proportional to the number of patients in the study and normalized to sum to 1.^16^^,^^17^ Regarding comparisons in the first-line setting with palbociclib+AI, we conducted a pooled analysis of mPFS and mOS from the PALOMA-1 and PALOMA-2 trials to establish a single 95% confidence interval for RCTs. Studies with fewer than 50 patients and/or follow-up before 20 months were excluded from the palbociclib+AI first-line analysis to reduce heterogeneity and strengthen pooled estimates. This additional filter was not applied to fulvestrant studies due to the limited number of RWSs and the shorter PFS benefit obtained with the palbociclib+fulvestrant combination.

We considered real-world estimates comparable to the PFS/OS of PALOMA RCTs if the pooled median or weighted median PFS/OS fell within the respective 95% confidence intervals of the RCTs. Similar criteria were applied to the pooled hazard ratios of PFS/OS for palbociclib+AI vs AI alone in both visceral, nonvisceral, and bone-only subgroups. A sensitivity analysis carried out by removing all studies with fewer than 100 patients and less than 24 months of follow-up was conducted for each endpoint whenever feasible (ie, at least 2 RWSs to estimate the median of medians and the weighted median PFS/OS). Only for comparisons with the PALOMA-3 trial, the duration of follow-up criteria was not applied as the pivotal trial itself presented a follow-up before 24 months.

The methodological index for non-randomized studies (MINORS) was used for the qualitative assessment, due to its ability to evaluate the methodological quality of single-arm studies.^18^ Statistical analyses were performed using R software (R Foundation for Statistical Computing, Vienna, Austria). Approximate 95% confidence intervals for pooled medians and weighted pooled medians were calculated in R using the *metamedian* package.

The study was registered on Open Science Framework, with DOI: 10.17605/OSF.IO/ZJD9T.

## Results

### Study characteristics

We identified 12 RWSs for a total of 4940 of patients.^19-30^ Trial selection is reported in Figure S1. The median follow-up for the studies included was 23.2 (interquartile range = 18.8-29.4) months. Median age was 63 years. Two studies (16.7%) enrolled only postmenopausal patients, and 10 (83.3%) enrolled pre- and postmenopausal patients. Among the included studies, 5 used palbociclib+AI exclusively in the first-line (41.7%), 3 employed palbociclib+AI in the first-line or later (25%), 1 study administered palbociclib+fulvestrant only in the first-line (8.3%), and another study used palbociclib+fulvestrant beyond the second-line (8.3%). Additionally, 5 studies enrolled patients who received treatment with palbociclib+AI or fulvestrant in both initial and subsequent lines of therapy (41.7%). Main details of the included studies are described in Table 1.

**Table 1.** Description of included studies’ main features.

Region	First author	Year	Study type	Menopausal status	Median age	Treatment	Setting	N	Median FU	Median PFS	Lower 95% CI	Upper 95% CI
United States/Canada	Tripathy D	2022	Observational, prospective	Pre and post	64	PAL+AI	1L	571	37.9	35.2	31.3	37.5
United States	Law JW	2020	Observational, retrospective	Pre (11%) and post (86%)	66	PAL+AI	1L	242	22.4	31.7	27.9	NR
United States	Rugo HS	2022	Observational, retrospective	Postmenopausal	67	PAL+AI	1L	1572	23.9	19.3	17.5	20.7
United States	Rugo HS	2023	Observational, retrospective	Postmenopausal and age ≥65	72	PAL+AI	1L	490	20.2	22.2	20	30.4
United Kingdom	Palmieri C	2023	Observational, retrospective	Pre (30%) and post (70%)	57	PAL+AI	1L	137	24	22.8	16.5	NR
Portugal	Alves da Costa F	2023	Observational, retrospective	Pre (33%) and post (67%)	58	PAL+AI	1L	131	28.3	19.5	14.2	24.2
United States	Patt D	2022	Observational, retrospective	Pre and post	65	PAL+AI	1L	813	20.2	20	17.3	22.2
Europe	Oikonomidou O	2023	Observational, retrospective	Pre (14.1%) and post (78.3%); 7.5% NA	64	PAL+AI	1L	668	32.7	31.8	27.7	35.4
United States	Varella^a^	2019	Observational, retrospective	Pre and post	54	PAL+F	1L	34	10.2	11.6	8.2	NR
Italy	Palumbo^a^	2021	Observational. prospective	Pre (27%) and post (73%)	62	PAL+F	1L	29	14.9	13.5	6.5	18
Netherland	Hackert	2023	Observational, retrospective	Pre and post	57	PAL+F	≥2L	229	34.8	11.6	10.2	13.9
Asia	Lee	2021	Observational, retrospective	Pre (3%) and post (97%)	57	PAL+F	≥2L	24	14.6	6.4	5.33	NR

Region	Study name	Year	Study type	Menopausal status	Median age	Treatment	Setting	N	Median FU	Median PFS	Lower 95% CI	Upper 95% CI
Europe/North America/Asia	PALOMA-1/TRIO-18	2020	R 1:1, Open-label	Postmenopausal	63	PAL+AI	1L; PH2	84	64.7	20.2	13.8	27.5
Europe/North America/Asia	PALOMA-2	2022	R 2:1, Double-blinded	Postmenopausal	62	PAL+AI	1L; PH3	444	90.1	27.6	22.4	30.3
Europe/North America/Asia	PALOMA-3	2018	R 2:1, Double-blinded	Postmenopausal	57	PAL+F	≥1L	347	14	11.2	9.5	12.9

Region	First author	Year	Study type	Menopausal status	Median age	Treatment	Setting	N	Median FU	Median OS	Lower 95% CI	Upper 95% CI
United States/Canada	Tripathy D	2022	Observational, prospective	Pre and post	64	PAL+AI	1L	571	35.7	53.3	42.1	103.7
United States	Law JW	2020	Observational, retrospective	Pre (11%) and post (86%)	66	PAL+AI	1L	242	23.9	49.1	45.2	57.7
United States	Rugo HS	2022	Observational, retrospective	Postmenopausal	67	PAL+AI	1L	1572	23.9	NR	NR	NR

Region	Study name	Year	Study type	Menopausal status	Median age	Treatment	Setting	N	Median FU	Median OS	Lower 95% CI	Upper 95% CI
Europe/North America/Asia	PALOMA-1/TRIO-18	2020	R 1:1, Open-label	Postmenopausal	63	PAL+AI	1L; PH2	84	64.7	37.5	31.4	47.8
Europe/North America/Asia	PALOMA-2	2022	R 2:1, Double-blinded	Postmenopausal	62	PAL+AI	1L; PH3	444	90.1	53.8	49.8	59.2
Europe/North America/Asia	PALOMA-3	2018	R 2:1, Double-blinded	Postmenopausal	57	PAL+F	≥1L	347	73.3	34.8	28.8	39.9

Abbreviations: AI = aromatase inhibitor; CI = confidence interval; F = fulvestrant; FU = follow-up; L = treatment line; NA = not available; OS = overall survival; PAL = palbociclib; PFS = progression-free survival; PH = trial phase; R = randomized.

a Data analyzed also for second-line and beyond the second-line.

### PFS analysis

Eight studies (4624 patients) with palbociclib+AI as first-line therapy were included in the analysis. Median follow-up was 24 months (interquartile range = 20.75–31.6, min-max range = 20.2–37.9). Studies included were heterogeneous with regard to follow-up and sample size. Two studies (25%) enrolled only postmenopausal patients, and 6 (75%) enrolled pre- and postmenopausal patients.

**Figure 1.**
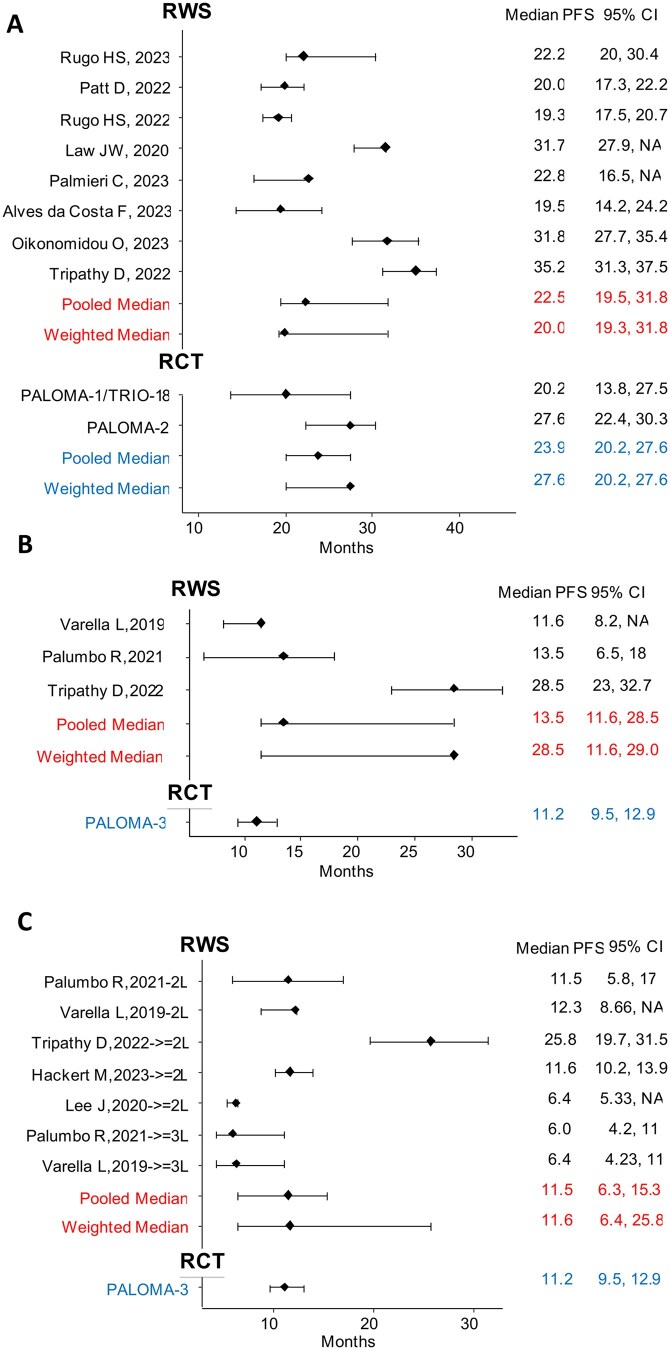
Pooled median and weighted median of first-line PFS and second-/further-line in RWSs: (**A**) first-line RWS of palbociclib+AI; (**B**) first-line RWS of palbociclib+fulvestrant; and (**C**) second-/further-line RWS of palbociclib+fulvestrant. AI = aromatase inhibitor; CI = confidence interval; L = line; PFS = progression-free survival; RWS = real-world studies.

The MMPFS across all RWSs was 22.5 months (95% CI = 19.5 to 31.8), similar to those of the PALOMA-1/TRIO-18 study, which reported a median PFS of 20.2 months (95% CI = 13.8 to 27.5), and PALOMA-2 (27.6 months, 95% CI = 22.4 to 30.3).^31^^,^^32^ Additionally, we estimated a pooled median of PALOMA-1/2 to obtain a single confidence interval for comparison, and the MMPFS of the RWSs was found to be comparable with that of the pooled median of the 2 RCTs PALOMA-1/2 (23.9, 95% CI = 20.2 to 27.6) (Figure 1, A). The WMPFS was 20.0 (95% CI = 19.3 to 31.8), comparable to the PALOMA-1 result but lower than that of PALOMA-2 and pooled PALOMA-1/2 PFS (27.6, 95% CI = 20.2 to 27.6) (Figure 1, A). Although all studies included at least 100 patients, 3 were removed for follow-up before 24 months and a sensitivity analysis was conducted. The resulting MMPFS was 22.8 (19.3 to 34.4), whereas the WMPFS was 19.7 (19.3 to 35.2). Results were consistent with the main analysis.

Three studies enrolled pre- and postmenopausal women treated in first-line with palbociclib+fulvestrant (372 participants).^19^^,^^20^^,^^26^ Two studies with significantly smaller sample sizes then PALOMA-3, reported mPFS quite consistent with the pivotal RCT, which reported a mPFS of 11.2 months (95% CI = 9.5-12.9)^33^ (Figure 1, B). In contrast, Tripathy’s study, with a larger sample size and longer follow-up duration, reported a higher mPFS than the other 2 studies.^19^ Its mPFS was 28.5 months (95% CI = 23-32.7), exceeding both the other studies and that of the PALOMA-3 trial. Overall, the MMPFS across all studies was 13.5 months (95% CI = 11.6-28.5), which was similar to the median PFS observed in the PALOMA-3 study. The WMPFS was 28.5 (95% CI = 11.6-29.0), higher than the PALOMA-3 result^33^ (Figure 1, B). When removing studies with <100 patients, only Tripathy et al remained, with results outperforming those of PALOMA 3.

Five studies were included in the analysis (564 patients) of second-/further-lines, with 2 of them reporting separate data for second-line and more advanced lines.^19^^,^^20^^,^^26^^,^^29^^,^^30^ The median PFS in the second-line was similar to that of PALOMA-3, which also included patients with AI-resistant disease treated in first-line.^33^ Real-world data beyond second-line were mostly worse; median PFS was 6.4 and 6 months, respectively. Only Tripathy’s study demonstrated a median PFS twice as long as PALOMA-3 (25.8 months; 95% CI = 19.7 to 31.5).^19^ Overall, the MMPFS was 11.5 months (95% CI = 6.5 to 15.3) and the WMPFS was 11.6 (95% CI = 6.4 to 25.8), both comparable to PALOMA-3 mPFS (11.2, 95% CI = 9.5 to 12.9).^33^ (Figure 1, C). In a sensitivity analysis where studies with sample sizes below 100 patients were excluded,^20^^,^^26^^,^^29^ the MMPFS was 18.7 (11.6 to 25.8) and the WMPFS 11.6 (11.6 to 25.8), supporting the main result.

### OS with palbociclib+AI or fulvestrant

**Figure 2.**
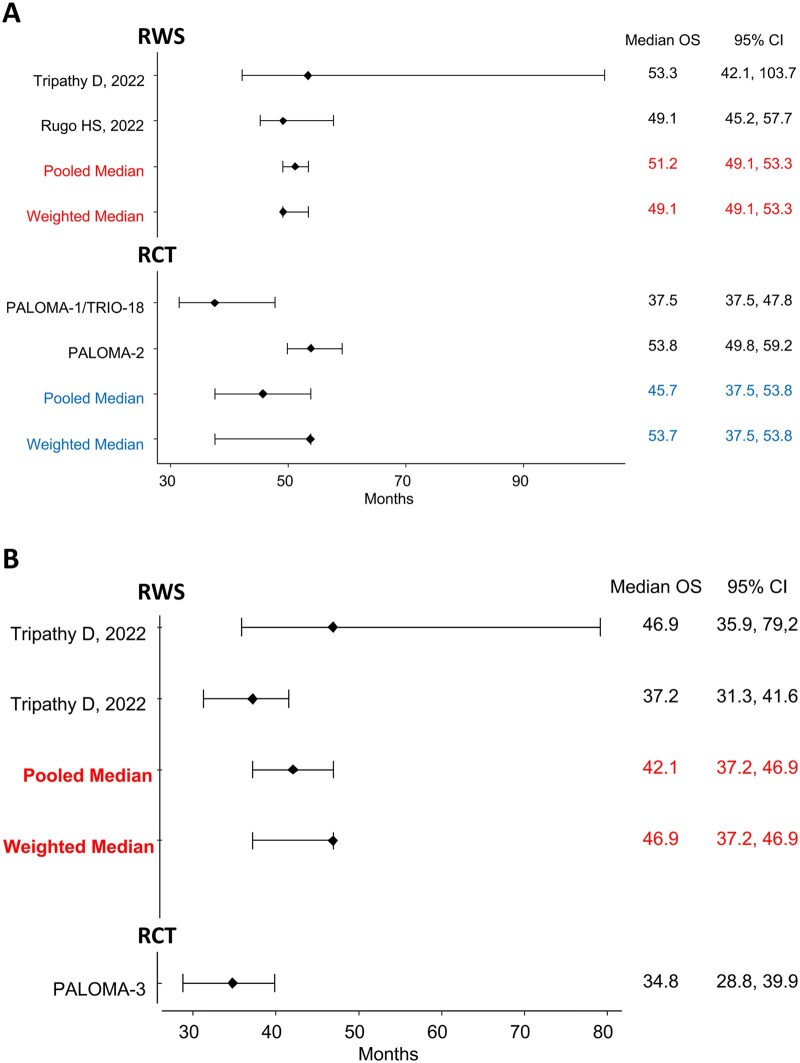
Pooled median and weighted median of first-line OS and second-/further-line in RWSs: (**A**) first-line RWS of palbociclib+AI; (**B**) first- and second-/further-line RWS of palbociclib+fulvestrant. AI = aromatase inhibitor; CI = confidence interval; OS = overall survival; RCT = randomized clinical trial; RWS = real-world studies.

Two studies (2143 patients) provided mOS data for patients treated with palbociclib+AI as first-line therapy.^19^^,^^23^ The MMOS was 51.2 months (95% CI = 49.1 to 53.3), and the WMOS was 49.1 months (95% CI = 49.1 to 53.3). These results are better than those observed in PALOMA-1 (37.5 months, 95% CI = 37.5 to 47.8) but comparable to PALOMA-2 (53.8 months, 95% CI = 49.8 to 59.2).^34^^,^^35^ The MMOS of the RWSs proved to be slightly superior to the pooled mOS of the PALOMA-1/2 studies (45.7 months, 95% CI = 37.5 to 53.8), whereas the WMOS was similar to that of the RCTs (53.7 months, 95% CI = 37.5 to 53.8) (Figure 2, A).

Only 1 study reported mOS data for patients receiving palbociclib+fulvestrant as first- and second-/further-line, with 309 and 177 patients, respectively.^19^ In the first-line treatment group, the mOS was 46.9 months (95% CI = 35.9 to 79.2), better than that of PALOMA-3 (mOS = 34.8; 95% CI = 28.8 to 39.9).^36^ Conversely, the mOS value for the second-line treatment group, at 37.2 months (95% CI = 31.3 to 41.6), was more consistent with that of PALOMA-3. When pooling the results of first and further lines (as in the PALOMA-3), the MMOS and WMOS were 42.1 (95% CI = 37.2 to 46.9) and 46.9 months (95% CI = 37.2 to 46.9), respectively (Figure 2, B), slightly better than the mOS observed in the RCT.

No sensitivity analyses were conducted for the OS endpoint, as both included RWSs recruited more than 100 patients and had a follow-up of 24 months or more.

### Real-world palbociclib efficacy according to visceral involvement

Three studies reported survival outcomes stratified by visceral/nonvisceral metastasis in terms of PFS^20^^,^^21^^,^^25^ and only 1 of those reported OS, as well (not shown).^20^ Heterogeneity in treatment line and ET companion, definition of visceral/nonvisceral disease and comparisons provided prevented us from deriving reliable pooled estimates (Supplementary Methods). Hence, a description of the outcomes in visceral and nonvisceral disease according to each study is reported in Table S1.

**Figure 3.**
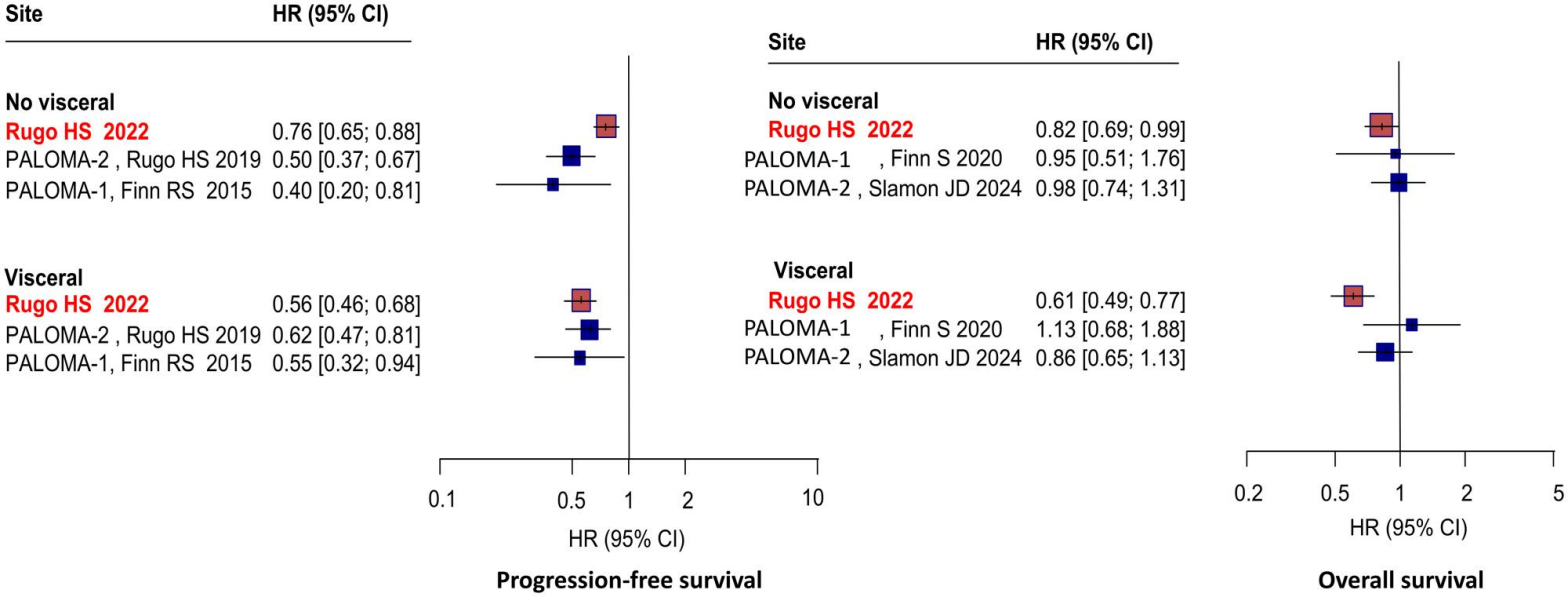
Progression-free survival and overall survival in patients with visceral/nonvisceral disease treated with P+AI vs AI. AI = aromatase inhibitor; CI = confidence interval; HR = hazard ratio.

Only Rugo et al. provided a real-world comparison between palbociclib-treated vs untreated patients according to visceral metastasis status, exclusively in the first-line and only with AI as endocrine treatment.^23^ In the subgroup with visceral metastases, palbociclib+AI showed superior PFS than AI (HR = 0.56, 95% CI = 0.46 to 0.68), in line with the results of PALOMA-2 (HR = 0.62, 95% CI = 0.47 to 0.81) and PALOMA-1 (HR = 0.55, 95% CI = 0.32 to 0.94).^32^^,^^37^ In the subgroup without visceral metastases, the combination provided a PFS benefit. The relative advantage in the palbociclib+AI group appeared to be more pronounced in the PALOMA-2 (HR = 0.50, 95% CI = 0.37 to 0.62) and PALOMA-1 (HR = 0.40, 95% CI = 0.20 to 0.81) than in real-life (HR = 0.76, 95% CI = 0.65 to 0.88) (Figure 3). Similarly, in bone-only disease the subgroup analysis showed a greater advantage of the palbociclib+AI combination in PALOMA-2 (HR = 0.41, 95% CI = 0.26 to 0.43) and PALOMA-1 (HR = 0.29, 95% CI = 0.09 to 0.95) compared with that obtained in the RWS study (HR = 0.74, 95% CI = 0.62 to 0.88).

In patients with visceral disease, the combination of palbociclib+AI showed superior OS than AI in the real-world setting (HR = 0.61, 95% CI = 0.49 to 0.77) with a better performance than that observed in the PALOMA-2 (HR = 0.86, 95% CI = 0.65 to 1.13) and PALOMA-1 (HR = 1.13, 95% CI = 0.68 to 1.88) trials.^23^^,^^34^^,^^35^ In the subgroup without visceral disease, a beneficial effect in OS for the combination vs AI OS was still observed in real life, likely more pronounced than in PALOMA-1 (HR = 0.95, 95% CI = 0.51 to 1.76) and PALOMA-2 (HR = 0.98, 95% CI = 0.74 to 1.31) (Figure 3). The result was similar in the real-world cohort with bone-only disease, where a significant benefit for the combination was still observed (HR = 0.77, 95% CI = 0.62 to 0.95) and in line with PALOMA-1 (HR = 0.36, 95% CI = 0.12 to 1.05) and PALOMA-2 (HR = 0.77, 95% CI = 0.51 to 1.17).

### Quality assessment

**Figure 4.**
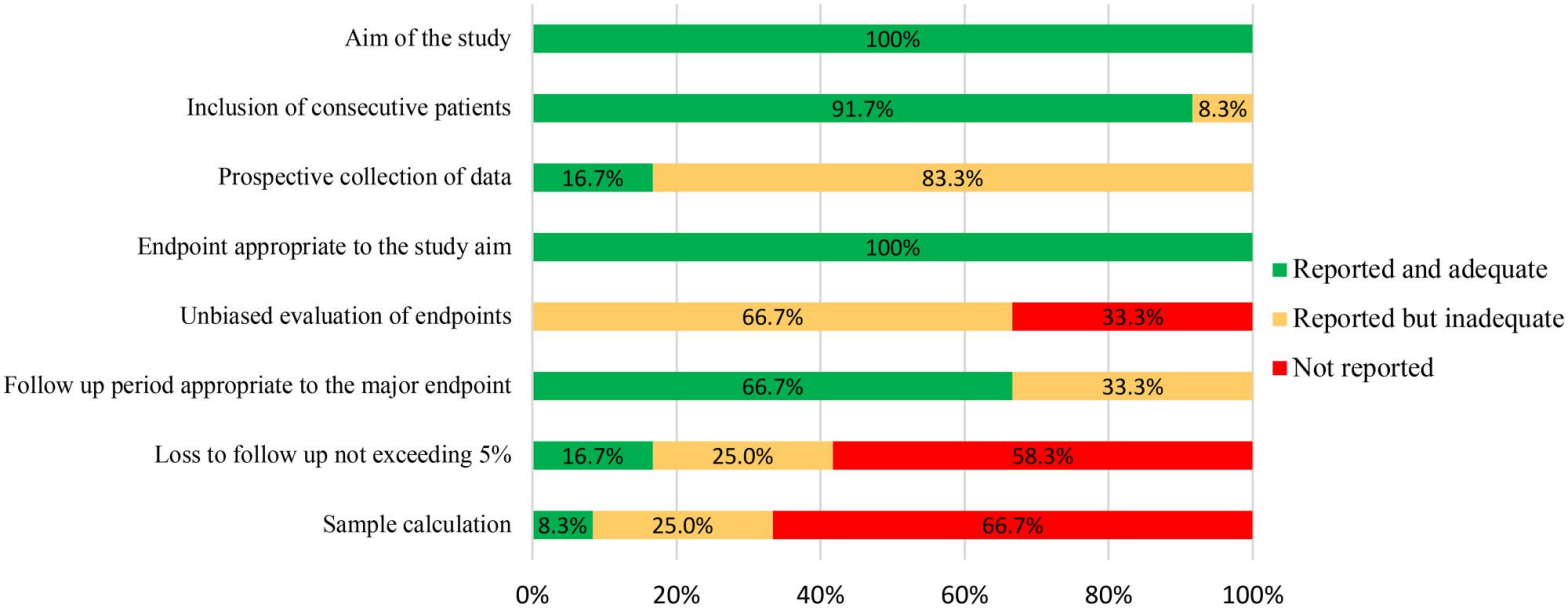
Risk of bias across studies assessed using the methodological index for non-randomized studies tool.

According to the MINORS scoring scale, only 1 study was of poor methodological quality,^26^ and the quality of the other 11 studies was moderate.^19-25^^,^^27-30^ Unbiased evaluation of endpoints and calculation of study size were not reported or were inadequate for most of the studies and loss to follow-up was lower than 5% only in 2 studies^21^^,^^24^ and not reported in 9 studies.^19^^,^^20^^,^^22^^,^^23^^,^^25-28^ All studies described clearly the aim and the endpoints. The scores are reported in Table S2 and evaluation outcomes are reported in Figure 4 and Figure S2.

## Discussion

The introduction of CDK4/6-inhibitors in combination with ET has significantly transformed the treatment landscape for HR+/HER2− MBC, establishing itself as a cornerstone in clinical management.^38^ Among these agents, palbociclib, the first CDK4/6-inhibitor approved by the U.S. Food and Drug Administration in 2015, has gained widespread use in clinical practice.^39^ Our meta-analysis has been conducted in response to the growing body of RWE assessing the efficacy and safety of palbociclib in daily practice. As RWE continues to expand, a comparative analysis between RCTs and RWE studies becomes increasingly crucial not only to validate the benefits observed in clinical trials but also to assess how these outcomes translate into routine clinical settings.^40^

We compared RWE with RCT results using the MM and WM methods to generate pooled real-world estimates of mPFS and mOS. These methods were adopted as they perform better than transformation-based approaches, in which the sample mean and its sampling variance are estimated from median data.^16^^,^^17^ We reviewed 12 RWSs involving patients with HR+/HER2− MBC who were treated with palbociclib in combination with either an AI or fulvestrant, administered as first-line or subsequent-line therapy. Overall, we observed a consistent palbociclib benefit across both RCTs and RWSs, reinforcing the efficacy of palbociclib in delaying disease progression both in first and further lines, as well as in combination with both AI and fulvestrant, respectively, in the first- and second-/further-line scenarios. Notably, regarding the combination with fulvestrant, the real-world study with the population features more closely resembling that of PALOMA-3 but set exclusively in the first-line scenario, reported a mPFS value double than that of the RCT, whereas the other 2 smaller first-line studies provided results more in line with those observed in the pivotal trial, which included mostly patients from the second-line.^19^^,^^20^^,^^26^^,^^33^ These results seem to support the use of AI as the ideal first-line ET partner, unless justified otherwise.

As expected, when observing individual RWSs results for patients who received palbociclib in combination with fulvestrant as third-line or beyond, the mPFS appeared to be shorter than what observed in previous lines.^19^^,^^20^^,^^26^^,^^29^^,^^30^^,^^33^ These findings highlight that while the benefit of palbociclib remains evident in later lines, its magnitude diminishes with increasing prior treatments. To note, among RWSs we also included results from the large POLARIS prospective observational trial.^19^ Additionally to its efficacy results in line with pooled estimates of PFS/OS in pivotal trials, the study recently provided insights into treatment persistence and patient-reported outcomes, reinforcing the benefit of palbociclib in a broader, less-selected patient population than in RCTs.^38^

Questions remain open regarding palbociclib OS impact in relation to other first-line CDK4/6-inhibitors, despite similar PFS benefit. MONALEESA-2, which enrolled postmenopausal women with HR+/HER2− recurrent or MBC who had not received prior systemic therapy for advanced disease, reported a significant benefit for ribociclib+letrozole compared to placebo+letrozole MONALEESA-2 demonstrated a statistically and clinically significant OS benefit,^39^ with comparable results also in the MONALEESA-7, where only premenopausal patients were included.^40^ A significant benefit was also observed with the combination with fulvestrant in the MONALEESA-3 RCT in AI-resistant MBC.^41^ Abemaciclib+AI in the first-line MONARCH-3 trial provided clinically meaningful OS improvement over AI alone, but the final OS result for the combination was not statistically significant,^42^ whereas the combination with fulvestrant in endocrine-resistant MBC was significantly superior to fulvestrant alone in the MONARCH-2.^43^ However, in terms of OS, none of the PALOMA trials showed a statistically significant superior benefit of palbociclib+ET over ET alone, despite clinically meaningful mOS improvements. It should be noted that these RCTs were not powered to detect a statistically significant OS advantage over the standard comparator, and an extensive and disproportionate censoring of patients with missing survival data in the PALOMA-2 might have also affected the OS comparison.^33^^,^^34^ Also, differences in the proportion of nonvisceral/bone-only disease (slightly higher in the PALOMA-2/3 than in the others), previous chemotherapy administration in the metastatic scenario (approximately 14% in the MONALEESA-7, more than 30% in the PALOMA-3, and none in the others),^40^^,^^44^ as well as possibly different post-progression treatments might have also contributed to final OS discrepancies. At the same time, it could be speculated that biochemical structural similarities between ribociclib and palbociclib but overall higher dosing for the former and shorter palbociclib half-life might have some impact on clinical outcomes.^45^ Similarly, the more potent activity against CDK4 of ribociclib/abemaciclib, against CDK2 for abemaciclib and its continuous administration might be additional factors contributing to the different OS results observed.^45^ Another possibility is the differential activity of ribociclib and palbociclib against the HER2-enriched intrinsic subtype.^46^ Ribociclib+ET showed consistent benefit in PFS and OS in a pooled analysis of the MONALEESA trials, whereas palbociclib+ET provided less compelling efficacy data in the nonluminal subtypes in both PALOMA-2 and -3 trials.^47-50^ Unfortunately, comparable analyses with abemaciclib have not been reported. That said, nonluminal subtypes tend to be infrequent in HR+/HER2− disease,^7^^,^^51^^,^^52^ making unlikely that OS differences should be at least entirely attributed to this aspect. To note, in the recently published PALMARES-2 RWS comparing palbociclib, ribociclib and abemaciclib in a large prospective cohort, abemaciclib and ribociclib showed a slightly better real-world OS than palbociclib, but OS data are still immature (23.4% of planned events) and the study was not randomized.^53^ In any case, whether or not the differences in OS across pivotal trials of CDK4/6-inhibitors reflect a true differential effect of the individual molecules, it is unlikely that head-to-head randomized comparisons will clarify this issue. To note, our real-world pooled OS estimates with palbociclib+AI were comparable to what observed in the PALOMA-2, and superior to mOS data shown in the PALOMA-1, suggesting that real-world populations may derive a greater OS benefit than initially observed in RCTs. Regarding the combination with fulvestrant, our pooled estimates also were superior to the mOS of PALOMA-3. Notably, the Triphathy’s RWS included a greater proportion of patients treated in the first-line in comparison to PALOMA-3, which might have particularly affected this specific comparison.^19^^,^^36^ Noteworthy, Rugo et al analyzed the comparative OS of CDK4/6-inhibitors plus an AI in HR+/HER2− MBC in a US real-world setting, highlighting important differences among available CDK4/6-inhibitors. The real-world median OS observed for palbociclib+AI was 49.1 months (95% CI = 45.2 to 57.7), comparable to the pooled PALOMA-1/2 data estimated in our study and very close to the PALOMA-2 individual result (53.8 months, 95% CI = 49.8 to 59.2), reinforcing the efficacy of this treatment approach. However, Rugo et al. also observed notable differences in OS among different CDK4/6 inhibitors, which may inform future treatment decisions, especially if further confirmed in the PALMARES-2 when mature OS data are released.^53^^,^^54^

Patients with visceral metastases pose a significant clinical challenge, given their typically poorer prognosis compared to those without visceral involvement. Reassuringly, our RWE with palbociclib aligns with RCTs in showing a significant benefit for the combination with both AI and fulvestrant.

For instance, among patients with visceral metastases, we observed a 44% risk reduction of progression in the palbociclib + AI group compared with the AI alone group, with a hazard ratio of 0.56 in RWS. Furthermore, when comparing OS between patients with visceral metastases, we noted a 39% reduction in the risk of death, with a hazard ratio of 0.61 in RWSs. This result surpassed that observed in PALOMA-2, where the hazard ratio was 0.86, and significantly outperformed PALOMA-1, where the hazard ratio was 1.13 and nonsignificant. However, when considering patients without visceral metastases, RCT PFS data showed a more profound benefit for the combination of palbociclib+ET than RWE. Conversely, regarding OS in patients without visceral metastases, the RWSs reported a hazard ratio of 0.82, reflecting a greater reduction in mortality risk compared to the PALOMA-1/2.^23^^,^^34^^,^^35^ Finally, we examined the subgroups of patients with bone-only disease and found that the benefit of the combination remained consistent across all studies, although with a greater reduction in risk seen in the PALOMA-1 and PALOMA-2 compared to the RWSs. Regarding OS, RWSs showed a benefit comparable to that of PALOMA-2 but inferior to PALOMA-1. However, the differences in hazard ratios between RWSs and RCTs can be attributed to variations in study population, study methodology, and follow-up. PALOMA-1 had a much smaller sample size compared with PALOMA-2 and the study by Rugo et al. That being said, RWSs may better reflect everyday clinical practice and include a more heterogeneous population compared with RCTs, which could influence differences in outcomes. To note, the PALMARES-2 showed comparable efficacy in the bone-only disease for the 3 CDK4/6-inhibitors, supporting the efficacy of palbociclib in this clinical subset beyond RCTs.^53^

Several limitations should be considered in interpreting these results: (1) the study heterogeneity including studies differed in sample size, follow-up duration, and treatment lines, which may influence pooled estimates; (2) real-world data variability, as the RWE studies primarily rely on retrospective data, potentially introducing selection bias and confounding variables; (3) the limited OS data available: only 2 RWE studies provided OS data,^15^^,^^19^ limiting direct comparisons with RCTs; (4) the subgroup inconsistencies: in visceral vs nonvisceral subgroup analyses, variations in definition criteria and patient populations may have influenced observed differences; and (5) we did not include the large real-world PALBOSPAIN study, or the previously mentioned PALMARES-2, because separate data according to a different ET backbone were not provided.^53^^,^^55^ Importantly, whenever feasible, sensitivity analyses were conducted to reduce RWSs’ heterogeneity and increase their reliability (long follow-up and considerable number of patients). These analyses supported main results.

Overall, our findings underscore the importance of bridging clinical trial data with real-world practice, recognizing that differences in patient populations, treatment adherence, and clinical management may influence outcomes. Future studies integrating prospective real-world data and longer follow-up durations will be essential in refining our understanding of the true clinical benefit of palbociclib in comparison to other CDK4/6-inhibitors, especially with regard to OS benefit. Importantly, the combination of palbociclib, fulvestrant, and PI3K-inhibitor inavolisib in HR+/HER2− MBC carrying *PI3KCA* mutations rapidly progressing on adjuvant AI has become the new standard treatment, following positive results from the Inavo120 RCT^56^ and posterior regulatory approvals. In this study, a significant OS benefit was also recently observed,^57^ making palbociclib the current standard-of-care CDK4/6-inhibitor for this specific subset of poor-prognostic patients.

## Supplementary Material

pkaf083_Supplementary_Data

## Data Availability

The data to generate this study’s results are available in the respective publications.
