# Supplementary material for: Comparative efficacy between real-world and randomized studies of palbociclib+endocrine therapy in HR-positive/HER2-negative metastatic breast cancer: systematic review and meta-analysis
Source: JNCI Cancer Spectr. 2025 Aug 12;9(5):pkaf083. doi: 10.1093/jncics/pkaf083 (PMC12413244; doi:10.1093/jncics/pkaf083)
Supplement: pkaf083_Supplementary_Data [file pkaf083_supplementary_data.pdf]

Supplementary Material

Supplementary methods 2

Search query 2

Methodology of study quality assessment 3

Detailed reasons to not report visceral vs. non visceral comparisons 4

References 5

Supplementary tables 6

Table S1. Studies providing results according to metastatic visceral involvement 6

Table S2. Quality assessment of studies according to MINORS score* 7

Supplementary figures 10

Figure S1. PRISMA flow-chart for study selection 10

Figure S2. Risk of bias in each study assessed using the MINORS tool 11

Supplementary methods

Search query

PubMed full search
**Filters**
English articles. Real-world studies. Period: 2019-2024
**Search details**
("Breast Neoplasms"[Mesh] OR "breast cancer" OR "Breast Neoplasm" OR "breast malignan" OR "breast tumor" OR "Mammary Cancer" OR "Mammary Neoplasm" OR "Breast Carcinoma" OR "Mammary Carcinoma" OR "Tumor of Breast" OR "Tumor of the Breast" OR "Cancer of Breast" OR "Carcinoma of Breast" OR "cancer of the breast"OR "carcinoma of the breast") AND (Neoplasm Metastasis[Mesh] OR metasta OR advanced[Title/abstract]) AND ("palbociclib" [Supplementary Concept] OR Cyclin-Dependent Kinase Inhibitor Proteins[MH] OR palbociclib OR "ibrance" OR "pd 0332991" OR "pd0332991" OR ("Cyclin-Dependent Kinase 4/antagonists and inhibitors"[Mesh] AND "Cyclin-Dependent Kinase 6/antagonists and inhibitors"[Mesh]) OR "cyclin dependent kinase 4 6 inhibitor"OR "cyclin dependent kinase 4 6 inhibitors"OR "cdk 4 6 inhibitor" OR "cyclin dependent 4 6 inhibitors" OR "cyclin dependent 4 6 kinase inhibitors" OR "inhibitor of cyclin dependent kinase 4a p16ink4a" OR "CDK4-6 inhibitor") AND ("Aromatase Inhibitors" [Pharmacological Action] OR "Aromatase Inhibitors"[Mesh] OR "Aromatase Inhibitor" OR "Inhibitor Aromatase") AND ((("ER+" OR "HR+" OR "ER POSITIVE" OR "hr positive" OR "estrogen receptor positive" OR "hormone receptor positive" OR "oestrogen receptor positive") AND ("her2 negative" OR "human epidermal growth factor receptor 2 negative" OR "her2 negative" OR "her2-" OR "human epidermal growth factor receptor 2 negative")) OR "HR+/HER2-" OR "ER+/HER2-" OR "HoR-positive/HER2-") AND ("REAL WORLD" OR RWD OR RWE OR "real life" OR "electronic health record" OR "Electronic Health Records"[Mesh] OR "Registries"[Mesh] OR "cancer registry" OR "cancer registries" OR "clinical outcome" OR "treatment outcome" OR Treatment Outcome[Mesh])

Methodology of study quality assessment

The Methodological Index for Non-Randomized studies (MINORS) was used for the qualitative assessment, due to its ability to evaluate the methodological quality of single arm studies. The MINORS tool is composed of eight items for non-comparative studies[1]: items are scored as 0 (not reported), 1 (reported but inadequate) or 2 (reported and adequate) with a global ideal score of 16. In this metanalysis, studies with total score ≤8 were considered poor quality, 9−12 moderate quality, and ≥13 good quality. Items 1 to 8 are as follows (1): a clearly stated aim (2); inclusion of consecutive patients (3); prospective collection of data (4); endpoints appropriate to the aim of the study (5); unbiased assessment of the study endpoint (6); follow-up period appropriate to the aim of the study (7); loss to follow up less than 5% (8); calculation of the study size.

Detailed reasons to not report visceral vs. non visceral comparisons

The real-world studies (RWSs) that reported outcomes for visceral vs. non-visceral/bone-only disease were those by Alves da Costa et al. (2023), Law et al. (2022), and Palumbo et al. (2021). However, heterogeneity in treatment line and endocrine treatment companion, definition of visceral/non-visceral disease and comparisons provided prevented us from deriving reliable pooled estimates. In Alves da Costa (2023), visceral disease was defined as involvement of lung/pleura, liver, peritoneum, or brain. Bone-only disease was categorized separately from non-visceral disease, which included all cases not falling into the visceral or bone-only categories. This study presented comparisons of visceral vs. bone-only and non-visceral vs. bone-only disease. In Law (2022), visceral disease included liver, lung, peritoneum, and pleural nodules. Non-visceral disease was not analyzed separately, while bone-only and bone plus other sites were grouped together. Only median real-world progression-free survival (rwPFS) was reported for the visceral and bone-only subgroups, with no formal statistical comparison. The study included patients treated in the first-line setting iwth palbociclib + aromatase inhibitors. In Palumbo (2021), visceral involvement was defined as the presence of metastases in visceral organs, including lung, liver, peritoneum, or pleura. Non-visceral disease was not explicitly defined, and it remained unclear whether bone-only cases were included in this group; however, a separate analysis for bone-only disease was presented. The study population included patients treated with fulvestrant or aromatase inhibitors across different lines of therapy. In this context, the visceral subgroup was compared to the non-visceral but not to the bone-only (reference category in Alves da Costa et al.).

References

1. Slim K., Nini E., Forestier D., Kwiatkowski F., Panis Y., Chipponi J. Methodological index for non-randomized studies (Minors): development and validation of a new instrument. ANZ J Surg. 2003;73:712–716. doi: 10.1046/j.1445-2197.2003.02748.x.

Supplementary tables

Table S1. Studies providing results according to metastatic visceral involvement

Metastatic sites	Study	Menopausal Status	Median age	Treatment	Line	Median FU (months)	N	Median PFS (months)	Lower 95% CI	Upper 95% CI
Visceral	Alves da Costa F, 2023	pre (33%) and post (67%)	58	PAL+AI	1L	28.3	72	15.5	11.2	23
	Law JW, 2022	pre (11%) and post (86%)	66	PAL+AI	1L	22.4	78	27.9	13.8	NA
	Palumbo R, 2021	pre (27%) and post (73%)	59	PAL+AI	≥1L	24	46	8.4	8.4	8.4
	Palumbo R, 2021	pre (27%) and post (73%)	64	PAL+F	≥1L	24	52	7.2	7.2	7.2
	PALOMA-3	pre and post	57	PAL+F	≥1L	-	206	9.2	7.5	11.1
	PALOMA-2	Post	62	PAL+AI	1L	37.6	214	19.3	16.4	24.2
Non-visceral	Alves da Costa F, 2023	pre (33%) and post (67%)	58	PAL+AI	1L	28.3	38	20.4	14.7	36.3
	Law JW, 2022	pre (11%) and post (86%)	66	PAL+AI	1L	22.4	123	44.9*	39.4	na
	Palumbo R, 2021	pre (27%) and post (73%)	59	PAL+AI	≥1L	24	44	13.7	13.7	13.7
	Palumbo R, 2021	pre (27%) and post (73%)	64	PAL+F	≥1L	24	40	14.5	14.5	14.5
	PALOMA-3	pre and post	57	PAL+F	≥1L	-	141	16.6	13.2	-
	PALOMA-2	Post	62	PAL+AI	1L	37.6	230	35.9	27.7	-

Legend. Pre: premenopause; post: postmenopause; PAL: palbociclib; AI: aromatase inhibitor; F: fulvestrant; FU: follow-up; L: line; PFS: progression-free survival; CI: confidence interval. *: bone-only.

Table S2. Quality assessment of studies according to MINORS score*

Author	Aim of the study	Inclusion of consecutive patients	Prospective collection of data	Endpoint appropriate to the study aim	Unbiased evaluation of endpoints	Follow up period appropriate to the major endpoint	Loss to follow up not exceeding 5%	Sample calculation	Score
Tripathy D	2 Reported and adequate	2 Reported and adequate	2 Reported and adequate	2 Reported and adequate	1 Based on individual treating physician	2 Reported and adequate	0 Not reported	1 It is reported as convenience sample	12/16
Law JW	2 Reported and adequate	2 Reported and adequate	1 Retrospective	2 Reported and adequate	1 Based on individual treating physician	2 Reported and adequate	2 Reported and adequate	0 Not reported	12/16
Rugo HS 2022	2 Reported and adequate	2 Reported and adequate	1 Retrospective	2 Reported and adequate	1 Based on individual treating physician	2 Reported and adequate	0 Not reported	2 Reported and adequate	12/16
Rugo HS 2023	2 Reported and adequate	2 Reported and adequate	1 Retrospective	2 Reported and adequate	1 Based on individual treating physician	2 Reported and adequate	0 Not reported	0 Not reported	10/16
Palmieri C	2 Reported and adequate	2 Reported and adequate	1 Retrospective	2 Reported and adequate	1 Based on individual treating	2 Reported and adequate	2 Reported and adequate	0 Not reported	13/16

					physician				
Alves da Costa F	2 Reported and adequate	2 Reported and adequate	1 Retrospective	2 Reported and adequate	0 Not reported	2 Reported and adequate	1 Reported 8.4% lost of follow-up	0 Not reported	10/16
Patt D	2 Reported and adequate	2 Reported and adequate	1 Retrospective	2 Reported and adequate	1 Based on individual treating physician	1 Median follow-up short	0 Not reported	1 Large sample but not reported statistical power	10/16
Oikonomidou O	2 Reported and adequate	1 not explicitly reported inclusion criteria	1 Retrospective	2 Reported and adequate	0 Not reported	2 Reported and adequate	0 Not reported	1 Large sample but not reported statistical power	9/16
Varella	2 Reported and adequate	2 Reported and adequate	1 Retrospective	2 Reported and adequate	0 Not reported	1 Median follow-up short	0 Not reported	0 Not reported	8/16
Palumbo	2 Reported and adequate	2 Reported and adequate	2 Reported and adequate	2 Reported and adequate	1 Based on individual treating physician	1 Median follow-up short	0 Not reported	0 Not reported	10/16
Hackert	2 Reported and adequate	2 Reported and adequate	1 Retrospective	2 Reported and adequate	0 Not reported	2 Reported and adequate	1 Not reported explicitly the % but discussed	0 Not reported	10/16
Lee	2 Reported and adequate	2 Reported and adequate	1 Retrospective	2 Reported and adequate	1 Based on individual treating	1 Median follow-up short	1 Not reported explicitly the %	0 Not reported	10/16

					physician		but discussed		

Footnotes. *Qualitative assessment was performed using the MINORS score. The eight items are scored 0 (not reported), 1 (reported but inadequate) or 2 (reported and adequate). The global ideal score being 16 for non-comparative studies. For the domains judged as 'reported but inadequate', the table provides a brief explanation of the detected reason for inadequacy.

Supplementary figures

Figure S1. PRISMA flow-chart for study selection

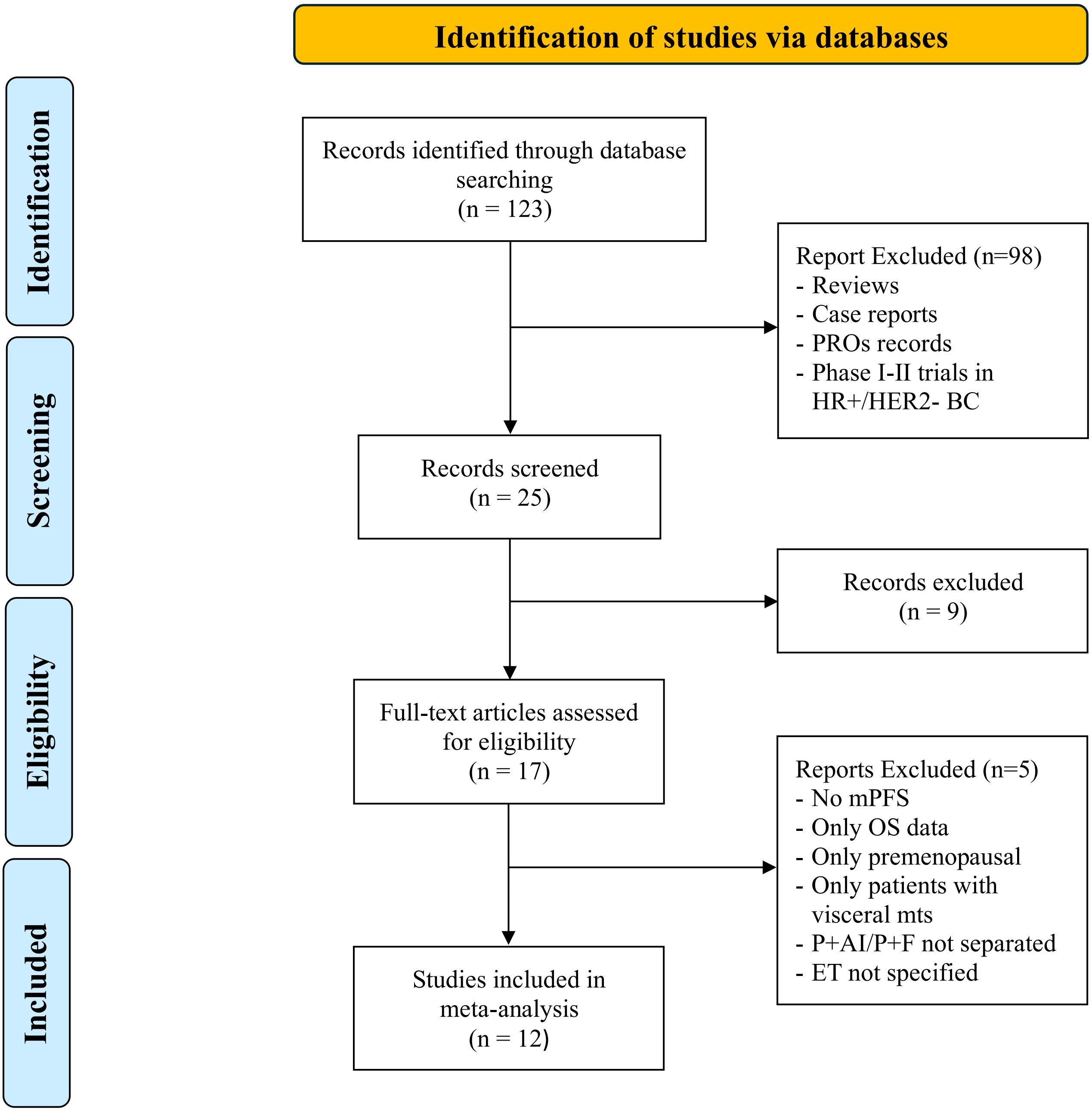

Legend. PRO: Patient-reported outcome; HR+/HER2-: Hormone receptor-positive/HER2-negative; BC: breast cancer; OS: overall survival; mPFS: median progression-free survival; P: palbociclib; AI: aromatase inhibitor; ET: endocrine therapy.

Figure S2. Risk of bias in each study assessed using the MINORS tool

Author	Aim of the study	Inclusion of consecutive patients	Prospective collection of data	Endpoint appropriate to the study aim	Unbiased evaluation of endpoints	Follow up period appropriate to the major endpoint	Loss to follow up not exceeding 5%	Sample calculation	Overall Score
Tripathy D 2022	2	2	2	2	1	2	0	1	12
Law JW 2020	2	2	1	2	1	2	2	0	12
Rugo HS 2022	2	2	1	2	1	2	0	2	12
Rugo HS 2023	2	2	1	2	1	2	0	0	10
Palmieri C 2023	2	2	1	2	1	2	2	0	12
Alves da Costa F 2023	2	2	1	2	0	2	1	0	10
Patt D 2022	2	2	1	2	1	1	0	1	10
Oikonomidou O 2023	2	1	1	2	0	2	0	1	9
Varella 2019	2	2	1	2	0	1	0	0	8
Palumbo 2021	2	2	2	2	1	1	0	0	10
Hackert 2023	2	2	1	2	0	2	1	0	10
Lee 2021	2	2	1	2	1	1	1	0	10
